# What Are the Ingredients for an Inequity Paradigm? Manipulating the Experimenter's Involvement in an Inequity Task with Dogs

**DOI:** 10.3389/fpsyg.2017.00270

**Published:** 2017-02-28

**Authors:** Désirée Brucks, Sarah Marshall-Pescini, Jennifer L. Essler, Jim McGetrick, Ludwig Huber, Friederike Range

**Affiliations:** Comparative Cognition Unit, Messerli Research Institute, University of Veterinary Medicine Vienna, Medical University of Vienna, University of ViennaVienna, Austria

**Keywords:** cooperation, inequity aversion, dogs, experimenter, competition

## Abstract

Cooperation is only beneficial if the outcome is equally shared between individuals involved in the cooperative interaction. A mechanism to limit the development of unequal cooperation is inequity aversion, the negative reaction to unequal treatment. While inequity aversion has been studied extensively across many animal species, it remains unclear whether inequity aversion elicited in experimental settings is directed to the cooperative partner animal or rather to the experimenter distributing the rewards unequally. In the current study we aimed to further investigate whether the presence of an experimenter distributing rewards is essential in order to elicit inequity aversion in dogs. We tested 22 dog dyads in an inequity task, requiring dyads to alternately press a buzzer in order to receive rewards of equal or unequal value. We manipulated the extent of the experimenter's involvement in the task: in the experimenter-present version an experimenter gave a command to the dogs to press the buzzer and delivered the rewards by pushing the bowls into the dogs' enclosure. In contrast, in the experimenter-absent version, no experimenter was visible and the buzzer and bowls were moved from behind a curtain. We found that dogs did not respond to the unequal treatment regardless of the experimenter's involvement in the task. Nonetheless, we found that dogs based their behavior on frustration and social facilitation in the experimenter-absent version of the task, suggesting that a social interaction with an experimenter may be one aspect necessary to elicit inequity aversion. One potential explanation for the absence of inequity aversion in the experimenter-present version of the task might be the reward delivery method. Using separate sets of reward bowls for each dog instead of a shared bowl could have removed a potentially important competitive aspect (i.e., shared resource) from the inequity paradigm. In addition, delivering the rewards via bowls, rather than directly handing the rewards to the dogs, might have caused dogs to perceive the task as less cooperative. These results suggest that both the presence of an experimenter causing inequity and the inclusion of a competitive or cooperative element in the task may be basic requirements for eliciting inequity aversion.

## Introduction

Cooperation is of clear advantage in order to achieve goals that cannot be accomplished alone. For example, hunting prey alone can be fruitless; however, cooperatively hunting with a partner, or in a group, can significantly increase hunting success, thereby resulting in a meal for each individual. Nonetheless, cooperation also entails competitive aspects. For example, following a successful hunt, competition arises over access to the carcass. However, there should be a balance between competition and cooperation; otherwise, cooperation might break down. It has, in fact, been shown that capuchin monkeys (Brosnan et al., 2006, 2010b) and chimpanzees (Melis et al., 2006a,b) prefer to cooperate with more tolerant partners and partners that share rewards equally, while cooperation ceased with partners that did not share equally. Consequently, cooperation is only beneficial for all individuals involved if the outcome of the joint action is equitable in the long-term. If one of the cooperating individuals does not receive an equitable share, it may be more beneficial for them to stop cooperating with these partners and to find new cooperative partners with a greater capacity to negotiate an equitable distribution of rewards. Inequity aversion has, therefore, been proposed to act as a mechanism to detect and respond to inequity, facilitating the search for partners that divide rewards more equitably and thereby stabilizing and increasing the benefit of cooperation over time (Fehr and Schmidt, 1999; Brosnan, 2011). Recent research has revealed that inequity aversion is much more common in the animal kingdom than previously thought with positive results in rats (Oberliessen et al., 2016), dogs (Range et al., 2009; Brucks et al., 2016), capuchin monkeys (Brosnan and de Waal, 2003), chimpanzees (Brosnan et al., 2005), and possibly crows and ravens (Wascher and Bugnyar, 2013).

Most studies investigating inequity aversion have used the so-called “exchange paradigm” as established in the original study investigating responses to unequal treatments in capuchin monkeys (Brosnan and de Waal, 2003). Typically, in the exchange paradigm, two monkeys sitting next to each other are required to alternately hand back tokens to the experimenter to obtain a reward. In the crucial inequity condition, the subject receives a lesser quality reward than the partner despite carrying out the same task (i.e., handing back the token to the experimenter. Interestingly, in order for an unequal reward distribution to elicit a response from a subject, the reward must follow an investment of effort in a task. For example, while capuchin monkeys showed no reaction to an unequal reward distribution if the rewards were handed over for “free” (i.e., without the need to perform a specific action) (e.g., Roma et al., 2006; Silberberg et al., 2009), they did show distinct reactions in studies using the exchange paradigm (e.g., Brosnan and de Waal, 2003; Fletcher, 2008), in which animals had to “work” to obtain a reward. Considering that captive animals are used to receiving unevenly distributed food rewards from caretakers without investing effort, but also without the option to influence this reward distribution, they might be more used to unequal treatment outside of a task context, thereby explaining the necessity of a task to elicit equity concerns (Brosnan et al., 2010a).

Given that dogs demonstrate cooperation with conspecifics as well as with humans (Ostojić and Clayton, 2014), but also that they demonstrate competitive behaviors (e.g., communal territorial defense: Bonanni et al., 2011), they represent an interesting model species to investigate inequity aversion outside the primate taxa. Importantly, dogs have been shown to react with aversion to inequity in a modified exchange task, in which they were alternately asked by an experimenter to give their paw (Range et al., 2009). Dogs refused to give their paw if they received nothing while their partner obtained a reward. In contrast, in a control condition, in which both animals received the same reward, dogs did not refuse to give their paw, thereby demonstrating that they are inequity averse. Importantly, the dogs stopped giving their paw earlier if they saw their partner receiving a reward while they were not rewarded, compared to when they were tested in an asocial control in which they, likewise, received no reward but no partner was present. Moreover, the subject was also less likely to follow the command if they were not rewarded but the partner was, compared to the condition in which both subject and partner were not rewarded. Interestingly, dogs did not stop the task if they received a reward of lower quality than their partner, in contrast to capuchins (e.g., Brosnan and de Waal, 2003) and chimpanzees (e.g., Brosnan et al., 2010a) who respond negatively to qualitatively inequitable food rewards. This might indicate that, to elicit inequity aversion in dogs, the differences in reward need to be more salient for (i.e., no reward for the subject but a reward for the partner).

While some studies investigated the outcome of cooperative interactions if the reward distribution was unequal (e.g., bar-pulling paradigm, Brosnan et al., 2006), or allowed the subjects to control the reward allocation (e.g., Proctor et al., 2013), most studies utilize simpler exchange-based tasks, which do not require the animals to interact with each other cooperatively. In these simple exchange-based tasks, animals are expected to respond to a command from the experimenter who then distributes the food. Consequently, it is the experimenter who distributes the rewards unequally with the conspecific partner having no direct involvement in the outcome. However, it remains unclear what the animals' perception of the human experimenter's involvement is. In a recent study we found that, following inequity conditions, dogs avoid both the experimenter who is responsible for the inequity, and the partner receiving the better reward, suggesting that both the human actor and the conspecific receiver are potentially perceived as cooperating partners in the interaction (Brucks et al., 2016).

Although dogs have been shown to exhibit inequity aversion, and to extend their aversion both to the experimenter causing the inequity and the partner dog involved in the situation, it is still not known what specific role the experimenter plays in a dog's reaction to inequity. While humans do not respond to unequal treatment if it is caused by a chance-based event such as a computerized task (Blount, 1995; Hachiga et al., 2009), or when the experimenter did not witness the effort invested prior to distributing rewards (Sloane and Premack, 2012), it is unclear whether the same rule applies to inequity aversion in non-human animals. Specifically, we do not know whether an experimenter needs to cause inequity by interacting with, and rewarding the dogs differentially, or whether the experimenter is perceived rather as a distributive entity and the mere fact of seeing the partner receiving a better reward is sufficient to elicit inequity aversion in dogs. Our aim in the current study was, therefore, to investigate whether the presence of an experimenter distributing the rewards is essential in order for dogs to respond negatively to inequity. In order to be able to run the same task in the presence but also in the absence of an experimenter, we designed a task comparable to the previous successfully used paw-giving paradigm (Range et al., 2009; Brucks et al., 2016). In this new inequity paradigm, dog dyads had to alternately press a buzzer with their paw in order to get access to rewards. We varied the involvement of the experimenter in the inequity task: in an *experimenter-present version* the dogs had to directly interact with an experimenter, whereas in the *experimenter-absent version*, no interaction with an experimenter was necessary. In the *experimenter-absent version* of the buzzer task, dogs were trained to press the buzzer as soon as it was moved into their enclosure; rewards were delivered from behind a curtain, which made the experimenter invisible to both dogs, via bowls connected to sticks. In contrast, in the *experimenter-present version* of the task, dogs were trained to press the buzzer on a verbal and gestural command given by a visible experimenter sitting in front of them; this experimenter delivered the food rewards by sliding them directly into the enclosure via bowls. In both versions of the task, the rewards obtained for pressing the buzzer differed across test conditions, with dogs either getting the same reward, one dog getting a better reward than the other, or one dog getting no reward while the partner is rewarded. In addition, as in Brucks et al. (2016), a food tolerance test was conducted directly following the inequity test, in order to investigate the influence of unequal treatment on subsequent social behaviors directed toward the partner dog.

Removing the experimenter from the task setup could potentially affect dogs' response to inequity in two ways. One possibility is that the interaction with the experimenter drives the response to inequity; thus, if we remove the experimenter from the setup, the response to inequity should diminish, with the task being perceived as more chance-based. In this case, dogs may base their refusals on individual reward expectations (contrast effect, Reynolds, 1961) rather than comparing outcomes with the partner. Additionally, we predict that dogs would no longer show changes in food tolerance toward the partner dog after unequal treatment (as was found in the experimenter-based paradigm, Brucks et al., 2016). Another possibility, however, is that the experimenter is not perceived by the dog as having a specific role in the inequity task and his/her presence is rather distracting the dogs from monitoring the partner's actions. If this were the case, we would expect dogs to pay more attention to their partner in the *experimenter-absent version*, and possibly, by attending more to the partner, also manifest their inequity aversion when qualitatively different rewards are delivered. In this case, after unequal test conditions (reward and quality inequity) dogs should show a reduced food tolerance toward the partner dog.

## Methods

### Subjects

Forty-four dogs (mean age: 5.0 ± 2.6 years.) of various breeds and mixes were tested in familiar dyads in this study (see Table S1). Only dogs living together in the same household for at least 1 year, without showing signs of food aggression, were included. All tests were conducted between January–October 2014 and April–July 2016 at the Clever Dog Lab in a test room (7 × 6 m). Ethical approval was obtained from the ethical commission of the University of Veterinary Medicine, Vienna (Approval number: 08/07/97/2013), and owners were required to sign a consent form prior to testing.

### Experimental procedures

Dogs were tested in familiar pairs using a between subject design. One experimental group participated in an *experimenter-present version* of the buzzer task (i.e., direct interaction with experimenter; *N* = 20 dogs), whereas the other group was tested in an *experimenter-absent version* of the buzzer task (i.e., no experimenter visible; *N* = 24 dogs; see Table S1 for individual characteristics of dogs). The experimenter was unfamiliar to the dogs. Twelve dyads (*experimenter-present version*: *N* = 8 dyads; *experimenter-absent version*: *N* = 4 dyads) already participated in another inequity aversion study (Brucks et al., 2016) using a different paradigm prior to participating in this study. Following each dyadic test condition a food tolerance test was conducted. The training procedure was identical for both tasks (see Table 1 for timetable of experimental procedure).

**Table 1:** **Experimental timetable for group of dogs in experimenter-present and -absent version of buzzer task**.

**Group**	**Training**			**Tests**	
	**Days 1–3**			**Days 4–11 (one test condition per day)**	
	**Food preference test**	**Bowl association**	**Buzzer training**	**Buzzer task**	**If social condition: Tolerance test**
Experimenter-present (*N* = 20 dogs)	Establish LVR and HVR	Associate reward types with bowl colors	Press buzzer on verbal + gestural command	Experimenter gives buzzer command and distributes rewards	Sharing of food between dogs following each dyadic condition
Experimenter-absent (*N* = 24 dogs)			Press buzzer when available in the enclosure	Buzzer and rewards are delivered covertly (i.e., no person in sight, no commands given)	

### Experimental setup

**Figure 1:**
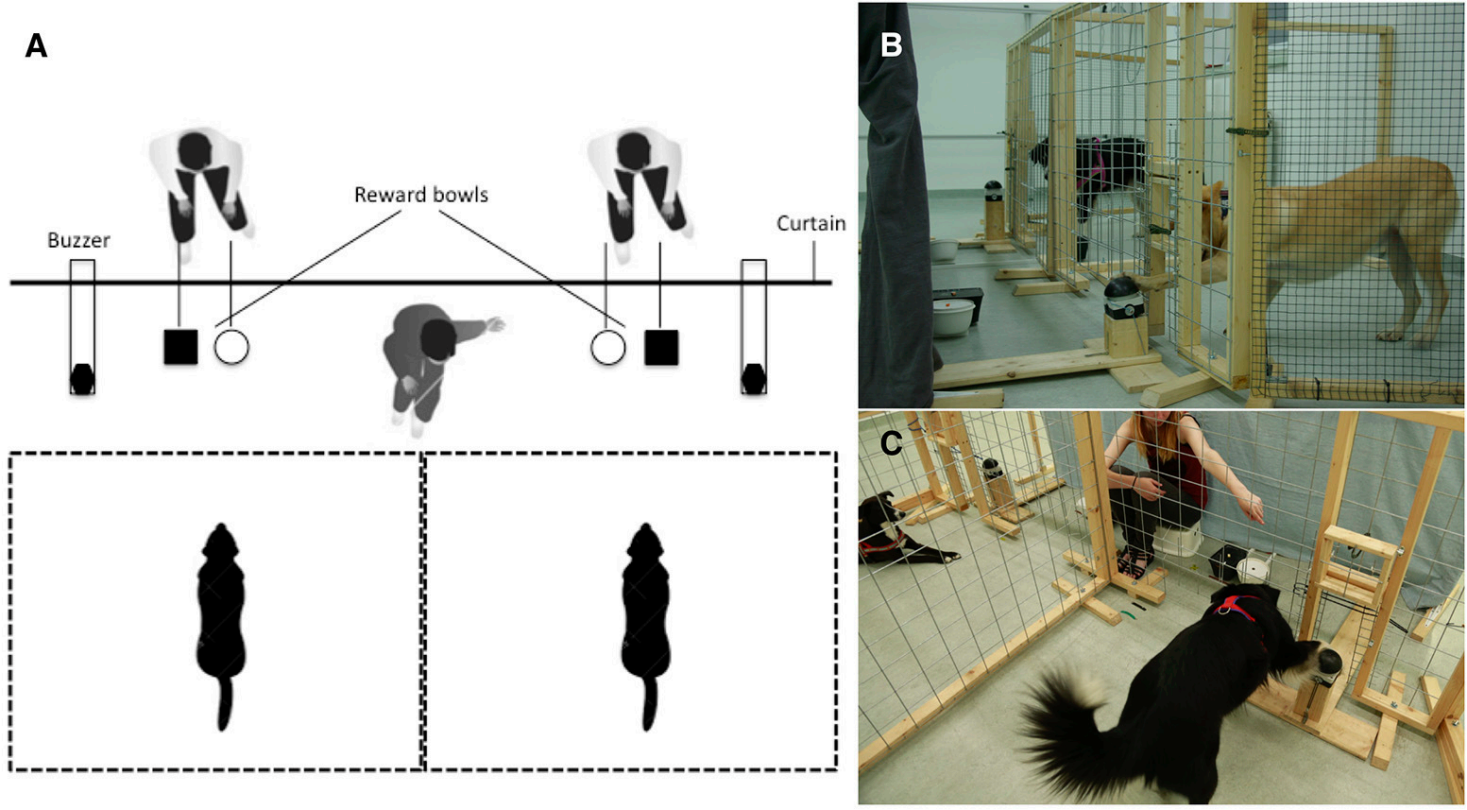
**Buzzer task setup for ***experimenter-present*** version: (A)** Sketch of buzzer setup from above: Buzzers and bowls are in the starting position, the two invisible experimenters are sitting behind a curtain, and the visible experimenter is sitting in front of the curtain (only in the experimenter-present version). **(B)** *Experimenter-absent* version: No experimenter is visible and the buzzers and bowls are moved from behind the curtain. The black dog (the partner) has just eaten its reward, while the buzzer is moved within reach of the subject (the fawn dog) who reaches out to press it. **(C)** *Experimenter-present* version: The visible experimenter is sitting in front of the curtain giving a verbal and gestural command to press the buzzer to the dog on the right (the partner), while the dog on the left (the subject) is waiting for its turn.

The apparatus consisted of two adjacent enclosures (2 × 2 m) covered by large-holed wire mesh (10 × 15 cm) attached to a wooden frame (see Figure 1A). The front fence had two openings on the ground level, one for the food bowls (40 × 20 cm) and one opening (25 × 45 cm) for the buzzer (Eaton® FAK-S/KC11/I). Given that the investment of effort has been shown to be essential for animals to respond to inequity (i.e., exchange token vs. reward for “free”), the buzzer was positioned on a wooden block to make the action more effortful for the dogs (see Figures 1B,C). Three different wooden blocks (sizes: 10, 25, and 40 cm) were used during testing depending on the dog's size and health condition [i.e., older dogs (>8 years of age) were given the smaller blocks]. During testing, both dogs were placed in the two separate enclosures (sides randomly assigned and counterbalanced across test sessions). There were always two bowls in front of each, one bowl for LVR and another bowl for HVR. Both food bowls were always baited and visible during testing. The rewards were placed on elevated lids (same color as the bowls) inside the bowls to enhance the visibility of rewards (see Figures 1B,C). The bowl with the HVR was always on the inner position (e.g., Figures 1B,C, black bowl contained HVR and white bowl LVR). The owner either left the room during testing (*N* = 13 dyads) or was hiding silently together with the two experimenters behind the curtain (*N* = 9 dyads). Two invisible and completely silent experimenters were always positioned behind an opaque curtain (see Figure 1A). Each experimenter was responsible for moving the buzzer and bowls for one dog. The dogs were observed by the experimenters via webcams; the view provided by the webcams allowed the experimenters to direct the buzzers through the openings. In the social version of the buzzer task, an additional visible experimenter was sitting in front of the curtain giving the commands to the dogs to press the buzzer and rewarding them for successful buzzer presses by moving the corresponding bowl into the dog's enclosure (see Figure 1C).

### Training

Prior to testing (see Table 1 for procedural overview), we conducted food preference tests with each dog to establish a low value reward (LVR) and a high value reward (HVR) (see Supplementary Material for details on procedure). Only reward types for which both dogs within a dyad had the same preference were used for the test. Following the preference test, dogs were trained to associate the two reward types with two bowls of different shape and color (see Supplementary Material for detailed descriptions). Additionally, the dogs were trained to press the buzzer with their paw using a secondary reinforcer and the LVR (see Supplementary Material for details).

### Test procedure

Each test session started with 5 *warm-up trials* in order to ensure that the dogs still remembered the task; the buzzer was moved into the enclosure and if the dog pressed it, one piece of LVR was presented on a plastic plate. A plate was used in order to make the *warm-up trials* as different as possible from test trials, in which the black and white bowls were used. The partner always completed the *warm-up trials* first, and then it was the subject's turn. If a dog refused to press the buzzer, another training session (see training procedure Supplementary Material) was conducted and the test was postponed until the next testing day. However, if the problem occurred again the next time, the dyad was excluded from the study (*N* = 1). If both dogs successfully completed the warm-up trials, the test trials started.

The general test procedure was identical for both versions of the buzzer task. The two bowls were baited behind the curtain (one piece of HVR/LVR was put in the corresponding bowl) and pushed in front of the curtain simultaneously but were kept out of reach of the dogs (35 cm away from the fence). The two buzzers remained in front of the buzzer openings, out of the reach of the dogs (20 cm away from the fence). This position of bowls and buzzers was the starting and end position for each trial (see Figure 1A). A trial started by moving a buzzer into an enclosure. In each trial the partner dog was given a command first, followed by the subject dog. When the buzzer was pressed, the dog was rewarded with either a piece of LVR or HVR (depending on the condition) from the bowl. Following this, the bowl and buzzer were pulled to the starting position. The subject dog's trial began by moving the buzzer into the subject's enclosure; the subject was rewarded for pressing the buzzer with a reward from one of the bowls. The reward in the bowl remained available in the enclosure for 2 s; if it was not consumed in this time, it was pulled out again and the next trial started. The two experimenters, invisible to the dogs, were always responsible for baiting the bowls, pushing them into the starting position, and pulling them back behind the curtain after a trial was finished. In addition these two experimenters were responsible for moving the buzzer in and out of the enclosures in both versions (experimenter-present and—absent) of the buzzer task. After the buzzer was pushed into an enclosure, it was left there for 2 s; if the dog did not press it, it was pulled back to the starting position and then pushed back into the enclosure again for a further 2 s. This process was repeated a maximum of 10 times. During the 5th repetition the out-of-sight experimenter knocked twice on the wooden panel attached to the buzzer to redirect the dog's attention toward the buzzer while it remained inside the enclosure. Following this, the remaining 5 repetitions were conducted as before (i.e., no knocking sounds). If the dog refused to press the buzzer after 10 repetitions, the test session was terminated.

In the *experimenter-present version* of the buzzer task, an unfamiliar experimenter was sitting in front of the curtain (see Figure 1C), clearly visible and equidistant from both dogs. The experimenter gave a specific buzzer command (e.g., “press,” “paw,” “step,” “up”; same command for both dogs in a dyad) combined with a pointing gesture (i.e., extending and raising the arm completely while holding the index finger outstretched; the right arm was used for the right enclosure and the left arm for the left enclosure) directed toward the buzzer (see Movie 1 for procedure). The pointing gesture stopped after 2 s and the arm was lowered back to the lap. Each time the buzzer was pushed into the enclosure, the experimenter gave the same command (gesture + word using the same tone of voice), always avoiding eye contact with the dogs and without saying or gesturing anything else except for the command. If the buzzer was pressed within 2 s, the experimenter visibly pushed the respective bowl into the enclosure (see Movie 1). The experimenter was responsible for giving the buzzer command and rewarding the dogs by pushing the bowls into the enclosures; apart from those actions, she remained silent and motionless.

In the *experimenter-absent version* of the buzzer task, the bowls and buzzers were moved via sticks so that even the experimenter's hands were out of sight of the dog (see Figure 1B, Movie 1).

### Test conditions

Six test conditions were conducted (see Table 2) with 60 trials per session alternating between subject and partner dog (i.e., 30 trials per dog). We conducted two inequity conditions, one in which the partner dog received a reward of higher quality than the subject dog (quality inequity) and one in which the subject did not receive a reward while the partner did (reward inequity). Additionally, in order to investigate how dogs react when their individual reward expectations are not met, we incorporated a contrast condition (food control, e.g., Brosnan et al., 2005; Brucks et al., 2016). In this condition, both dogs were first shown the HVR by moving the bowl closer to the enclosure without making it accessible to the dogs before pulling it back again and moving only the LVR bowl into the enclosure. In order to investigate how the behavior of dogs changes when they are rewarded unequally, a baseline condition, in which both dogs received the same LVR reward (equity condition), was carried out. Additionally, in order to assess whether dogs based their refusal on their own reward expectations rather than socially comparing outcomes, and to rule out social facilitation in the reward inequity condition (enhanced motivation to give paw due to mere presence of partner), we included an asocial baseline condition (no-reward condition), in which dogs received no reward for pressing the buzzer (as in the reward inequity condition); however, no partner dog was present in the adjacent enclosure. Moreover, another asocial condition (assessment control) was conducted to establish that each dog was sufficiently motivated to press the buzzer 30 times in a row. The dogs were randomly assigned to start as the subject or as the partner prior to testing. Each dog served as the partner and as the subject during the test; however, the roles were only reversed in the two last test sessions. The order of test conditions was semi-randomized with the exception that neither the reward inequity (RI) nor the asocial no-reward condition (NR) were tested in the first session to avoid animals becoming frustrated at the beginning of testing. Furthermore, the asocial control conditions were always tested in consecutive sessions starting with the assessment condition (AC) first. If a dog refused to continue during a test session, the buzzer-pressing behavior was reinforced again 5 times using the HVR after the entire test session was finished. Only one test condition was carried out per test day, and no more than three tests were carried out per week. There was at least a 1 day break between each test.

**Table 2:** **Test conditions for buzzer task (adapted from Brucks et al., **2016**)**.

**Condition**	**Subject**	**Partner**
**SOCIAL CONDITIONS**		
Equity (ET)	LVR	LVR
Quality Inequity (QI)	LVR	HVR
Reward Inequity (RI)	No reward	HVR
Food Control (FC)	HVR moved, LVR given	HVR moved, LVR given
**ASOCIAL CONDITIONS**		
Assessment Control (AC)	LVR	— ^*^
No-Reward Control (NR)	No reward	— ^*^

* *In the asocial conditions, the LVR bowl was moved into the empty enclosure first before the subject's trial started, in order to rule out the possibility that dogs just react to the movement of the food but the payoff of the partner (see also Range et al., 2009)*.

### Tolerance test

Directly following each social test condition a food tolerance test was conducted following the procedure of Brucks et al. (2016). The tolerance tests following the equity and food control conditions were analyzed for both dogs within a dyad, whereas the tests following the reward inequity and quality inequity conditions were analyzed only for the subject dog. Consequently, six tolerance tests were conducted per dyad. This tolerance test was included as, we observed in our previous study (Brucks et al., 2016), dogs did not show behavioral reactions to inequity if rewards differed in quality; however, they did show a reduced co-feeding behavior following the inequity test. This suggests that dogs responded differently following the inequity test even though they did not react to it during the test, potentially due to a lack of inhibitory control (i.e., they did not reject the inequitable outcomes). Based on these results, and in order to have an additional measure of dogs' social behavior following the inequity test, we incorporated this measure.

A bowl (20 cm diameter) filled with slices from a total of 1.5 sausages was shown to both dogs before the experimenters guided the dogs, by their collars, out of the enclosures. The bowl was placed on the ground equidistant from both dogs and they were released by the experimenters at the same time. We measured the social tolerance between dogs while feeding from the bowl. The tolerance test was not conducted for two of the dyads, as the owners were afraid the test situation would provoke a fight between their dogs.

### Data analyses

All tests were video recorded and then coded using the Solomon coder beta (© 2015 by András Péter; http://solomoncoder.com/). We coded the following variables during the test: number of times the buzzer was pressed (max. 30), number of buzzer prompts (i.e., number buzzer commands given/number of times buzzer was moved into and out of enclosure until dog pressed it or test was terminated), gaze duration to partner enclosure (i.e., turning the head toward the partner enclosure), and stress signals (i.e., any of the following: yawning, mouth-licking, scratching; see Table S2 for definitions for those behaviors). Since dogs only refused to eat a reward in 0.03% of trials, we could not run any statistics on this variable. In order to take into account the different lengths of sessions due to refusals, we calculated an average occurrence rate per trial for the variables gaze to partner enclosure and stress signals. In the tolerance test we analyzed the duration of co-feeding, which was defined as simultaneously feeding from the bowl (i.e., both heads are inside of the bowl), and feeding alone from bowl (only one dog's head is in the bowl).

Statistics were performed in R (R Core Team, 2014) using the package “lme4 (Version: 1-1.7)” (Bates et al., 2015). The number of buzzer presses across conditions was analyzed using generalized linear mixed models (GLMM) with a binomial error and logit-link function. The data set was split up into two sets: one including the dyadic conditions (i.e., ET, FC, QI, RI), and the other set included the unrewarded conditions (i.e., NR, RI). Dyad was entered as random effect for the dyadic conditions, and dog identity was entered as random effect for the unrewarded conditions. The response variable for the GLMMs consisted of the number of times the dogs had pressed the buzzer out of the total buzzer commands given. Additionally, we utilized linear mixed models for modeling normally distributed continuous variables [average gaze duration (cube-root transformed), average amount of stress behaviors (log-transformed), co-feeding and feeding alone (both reciprocal transformed)]. Task context (*experimenter-present/-absent*), condition (ET, FC, QI, RI, and NR) and condition order, and experience with inequity test (yes, no) were entered as fixed effects. Classical stepwise backward regression analyses with likelihood ratio tests (LRT) were used for retaining only significant factors in the final model. One person coded all the videos and two people each coded 20% of the videos (one coded the videos of the *experimenter-present version* and one coded the *experimenter-absent version*) Cohen's kappa for frequencies: times buzzer pressed (*exp.-absent version*: 0.98, *exp.-present version*: 1.00), buzzer prompts (*exp.-absent version*: 0.98, *exp.-present version*: 1.00), and stress signals (*exp.-absent version*: 0.71, *exp.-present version*: 0.73); intra-class correlation coefficient (consistency) for durations: gaze to partner (*exp.-absent version*: 0.79, *exp.-present version*: 0.85), co-feeding (*exp.-absent version*: 0.90, *exp.-present version*: 0.97), and feeding alone (*exp.-absent version*: 0.97, *exp.-present version*: 0.87).

## Results

### Trials completed across condition

The number of completed buzzer prompts was not affected by experience with inequity paradigms [experience × condition interaction: LRT: dyadic conditions: $\chi_{\left(3\right)}^{2}$ = 0.38, *p* = 0.945, unrewarded conditions: $\chi_{\left(1\right)}^{2}$ = 0.39, *p* = 0.531]. We found a condition × task context interaction and consequently analyzed the two versions of the buzzer task separately [LRT: dyadic conditions: $\chi_{\left(3\right)}^{2}$ = 9.10, *p* = 0.028; unrewarded condition: $\chi_{\left(1\right)}^{2}$ = 3.65, *p* = 0.056].

**Figure 2:**
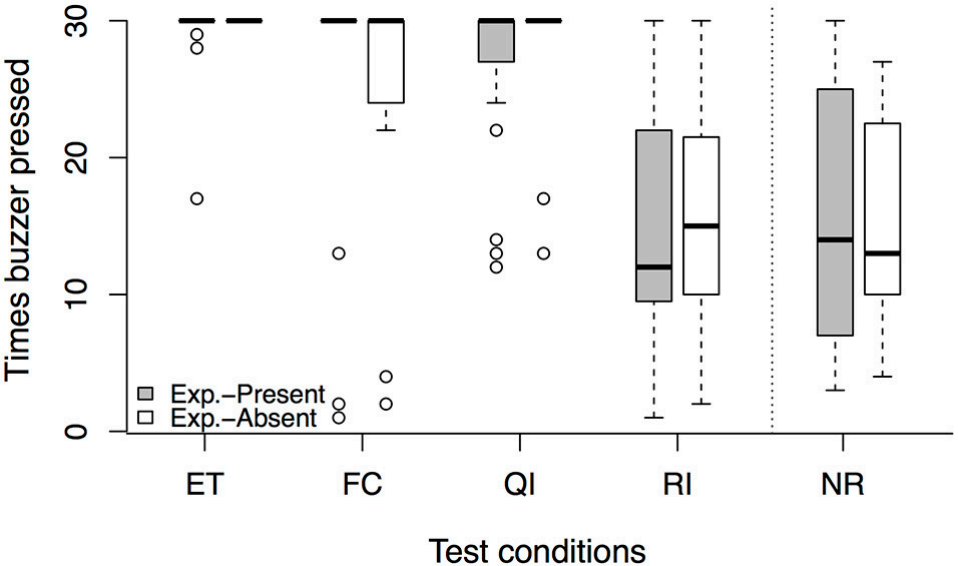
**Number of times the buzzer was pressed per test condition in the ***experimenter-present*** (gray) and ***experimenter-absent*** (white) version of the buzzer task**. Circles show outliers, black bars indicate median values, whiskers display upper and lower hinge, and boxes show the interquartile range. ET, equity; FC, food control; QI, quality inequity; RI, reward inequity; NR, no-reward control.

In the *experimenter-present buzzer task*, we found no session × condition interaction [LRT: $\chi_{\left(3\right)}^{2}$ = 0.81, *p* = 0.847]. However, there was an effect of condition on the number of completed trials in the dyadic conditions [LRT: $\chi_{\left(3\right)}^{2}$ = 107.27, *p* < 0.001]. Dogs pressed the buzzer less often in the RI compared to the ET condition (see Figure 2, Tables 3, 4). However, while again no session × condition interaction was observed [LRT: $\chi_{\left(1\right)}^{2}$ = 0.09, *p* = 0.764], we also found no significant difference between the RI condition and the NR control condition [LRT: $\chi_{\left(1\right)}^{2}$ = 0.47, *p* = 0.491, Table 3], indicating that the animal responded to not getting a reward rather than the inequity. Based on these results, no aversion to inequity emerged.

**Table 3:** **Average occurrence of buzzer presses, buzzer prompts, stress signals, and gaze durations per test condition for experimenter-present and experimenter-absent version of the buzzer task**.

	**Equity (ET)**		**Food control (FC)**		**Quality inequity (QI)**		**Reward inequity (RI)**		**No-reward control (NR)**	
	**Present**	**Absent**	**Present**	**Absent**	**Present**	**Absent**	**Present**	**Absent**	**Present**	**Absent**
Times buzzer pressed	28.6 ± 0.9	30.0 ± 0.0	25.5 ± 2.2	24.3 ± 2.0	26.8 ± 1.4	30.0 ± 1.0	14.5 ± 1.8	16.3 ± 1.7	15.6 ± 2.2	15.0 ± 1.4
Buzzer prompts	35.05 ± 1.4	36.8 ± 2.2	32.0 ± 2.9	36.7 ± 3.2	33.7 ± 1.1	33.0 ± 0.8	38.8 ± 3.3	37.1 ± 3.0	38.3 ± 3.9	40.8 ± 3.5
Stress signals per trial	0.1 ± 0.0	0.1 ± 0.0	0.3 ± 0.1	0.4 ± 0.1	0.3 ± 0.1	0.1 ± 0.0	0.8 ± 0.1	0.4 ± 0.1	0.4 ± 0.0	0.3 ± 0.1
Gaze duration to partner per trial	1.9 ± 0.4	2.2 ± 0.3	2.1 ± 0.4	4.7 ± 0.9	2.6 ± 0.5	2.3 ± 0.4	3.5 ± 0.5	4.4 ± 0.6	–	–

**Table 4:** **Summary of logistic regression model for experimenter-present and -absent buzzer task comparing number of buzzer pressed across conditions**.

**Condition**	***Experimenter-present version***		***Experimenter-absent version***	
	**Estimate ±*SE***	***p***	**Estimate ±*SE***	***p***
**ET BASELINE**				
FC	−0.07 ± 0.08	0.433	−0.22 ± 0.07	0.003^**^
QI	−0.03 ± 0.08	0.688	0.04 ± 0.07	0.569
RI	−0.83 ± 0.09	<0.001^**^	−0.63 ± 0.08	<0.001^**^
**NR BASELINE**				
RI	−0.07 ± 0.10	0.491	0.17 ± 0.08	0.045^*^

** *p < 0.005*,

* *p < 0.05*.

In the *experimenter-absent buzzer task*, likewise, we found no condition × session interaction [LRT: $\chi_{\left(3\right)}^{2}$ = 3.45, *p* = 0.328] but an effect of condition on the number of buzzer presses in the dyadic conditions [LRT: $\chi_{\left(3\right)}^{2}$ = 84.81, *p* < 0.001]. Dogs pressed the buzzer less often in the FC and RI conditions compared to the ET condition (see Figure 2, Tables 3, 4). In addition, condition had an effect on the number of buzzer prompts in the unrewarded conditions [LRT: $\chi_{\left(1\right)}^{2}$ = 4.00, *p* = 0.045]. Interestingly, dogs pressed the buzzer more often in the RI condition than in the NR control condition (see Figure 2, Tables 3, 4). And again, no condition × session interaction could be observed [LRT: $\chi_{\left(1\right)}^{2}$ = 0.00, *p* = 0.961], but no reaction to inequity could be observed.

### Stress behaviors

We found no task context × condition interaction on the average number of stress signals per trials [LRT: $\chi_{\left(3\right)}^{2}$ = 1.85, *p* = 0.605]; consequently, the two versions of the buzzer task were analyzed together. The average number of stress signals was not affected by test session [LRT: $\chi_{\left(3\right)}^{2}$ = 2.16, *p* = 0.539]. However, the dogs' stress levels were different across conditions [LRT: $\chi_{\left(3\right)}^{2}$ = 53.78, *p* < 0.001], and dogs exhibited more stress signals in the FC and RI conditions compared to the ET baseline level (see Tables 3, 5).

**Table 5:** **Summary of linear mixed model output for average stress signals per trial across dyadic conditions for both versions of the buzzer task**.

**Condition**	**Combined**	
	**Estimate * ± SE***	***p***
**ET BASELINE**		
FC	0.13 ± 0.05	0.007^**^
QI	0.02 ± 0.05	0.752
RI	0.31 ± 0.05	<0.001^**^

** *p < 0.005*.

**Figure 3:**
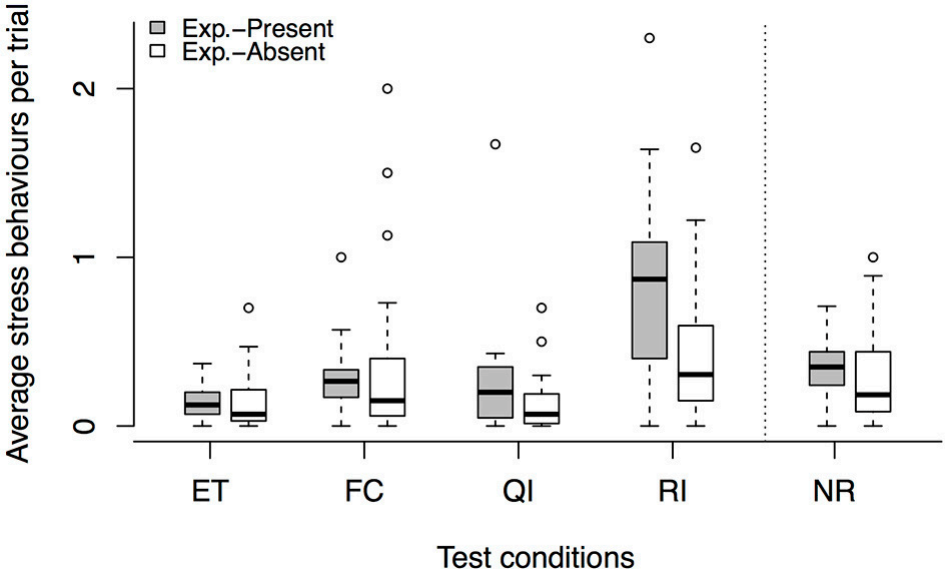
**Average number of stress signals per trial across conditions, comparing the ***experimenter-present*** (gray) with the ***experimenter-absent*** (white) buzzer task**. Circles show outliers, black bars indicate median values, whiskers display upper and lower hinge, and boxes show the interquartile range. ET, equity; FC, food control; QI, quality inequity; RI, reward inequity; NR, no-reward control.

For the unrewarded conditions, however, we found a condition × task context interaction [LRT: $\chi_{\left(1\right)}^{2}$ = 4.54, *p* = 0.033] on the number of stress signals per trial and analyzed the two versions of the buzzer task separately. In the *experimenter-present buzzer task*, we found that stress behaviors were affected by the condition [LRT: $\chi_{\left(1\right)}^{2}$ = 12.37, *p* < 0.001], which is not explained by a condition × session interaction [LRT: $\chi_{\left(1\right)}^{2}$ = 0.02, *p* = 0.877]. Dogs were more stressed in the RI condition compared to the NR condition, suggesting that there seems to be some sensitivity to the inequity. For the *experimenter-absent buzzer task*, we found an effect of condition [LRT: $\chi_{\left(1\right)}^{2}$ = 6.31, *p* = 0.012] but no condition × session interaction [LRT: $\chi_{\left(1\right)}^{2}$ = 0.13, *p* = 0.718] for the stress signals. Dogs showed more stress signals in the RI condition compared to the NR condition, again showing that the dogs potentially noticed the inequity (see Tables 3, 6). Overall, it seems that dogs were more stressed in the unrewarded conditions in the *experimenter-present* buzzer task compared to the *experimenter-absent* buzzer task (see Figure 3, Tables 3, 6).

**Table 6:** **Summary of linear mixed model output for average stress signals per trial across unrewarded conditions for experimenter-present and -absent versions of the buzzer task**.

**Condition**	***Experimenter-present version***		***Experimenter-absent version***	
	***Estimate ±* SE**	***p***	**Estimate * ± SE***	***p***
**NR BASELINE**				
RI	0.25 ± 0.07	0.002^*^	0.09 ± 0.04	0.020^**^

** *p < 0.005*,

* *p < 0.05*.

### Gazing behaviors

Since we found a task context × condition interaction on the average gazing behavior [LRT: $\chi_{\left(3\right)}^{2}$ = 9.22, *p* = 0.027], we split the data set up and analyzed the two versions of the task separately.

**Figure 4:**
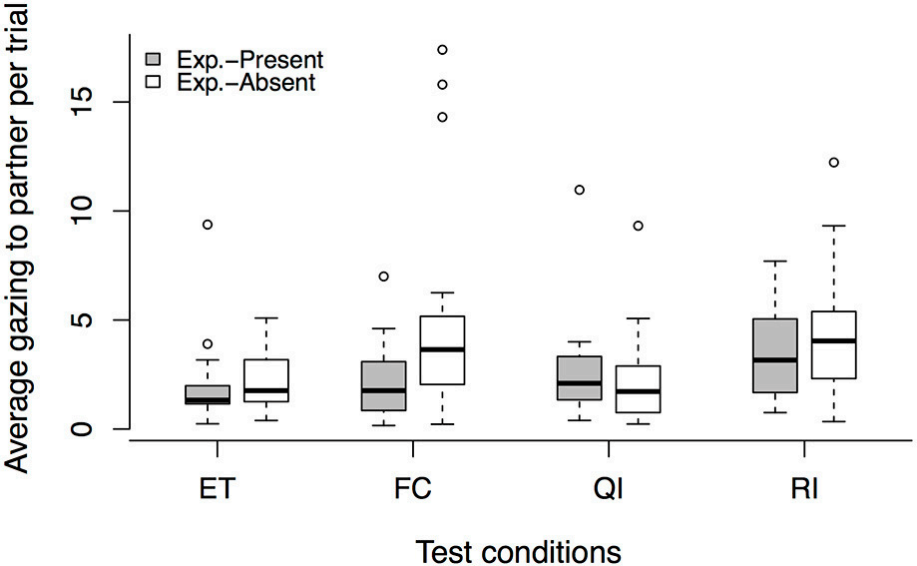
**Average gaze duration to partner enclosure per trial in each test condition, comparing the ***experimenter-present*** (gray) with the ***experimenter-absent*** (white) buzzer task**. Circles show outliers, black bars indicate median values, whiskers display upper and lower hinge, and boxes show the interquartile range. ET, equity; FC, food control; QI, quality inequity; RI, reward inequity; NR, no-reward control.

An effect of condition was found for the *experimenter-present buzzer task* [LRT: $\chi_{\left(3\right)}^{2}$ = 15.36, *p* = 0.002], but we found no order effect [condition × session interaction: LRT: $\chi_{\left(3\right)}^{2}$ = 1.44, *p* = 0.696]. Dogs gazed more toward their partner's enclosure in the RI condition compared to the ET baseline (see Figure 4, Tables 3, 7). For the QI condition we detected only a trend, with dogs gazing slightly more toward the partner than in the ET condition. Interestingly, we found that, in addition, those dogs that gazed more toward their partner's enclosure in the QI and RI conditions also refused to press the buzzer at some point (Spearman correlation: QI: *r*_*s*_ = −0.48, p = 0.033, RI: *r*_*s*_ = −0.64, *p* = 0.002).

**Table 7:** **Summary of linear mixed model comparing average gaze to partner's enclosure per trials across social conditions in experimenter-present and -absent version of the buzzer task**.

**Condition**	***Experimenter-present version***		***Experimenter-absent version***	
	**Estimate * ± SE***	***p***	**Estimate * ± SE***	***p***
**ET BASELINE**				
FC	0.05 ± 0.09	0.582	0.25 ± 0.10	0.015^*^
QI	0.16 ± 0.09	0.068	−0.05 ± 0.10	0.639
RI	0.31 ± 0.09	<0.001^**^	0.30 ± 0.10	0.004^**^

** *p < 0.005*,

* *p < 0.05*.

In the *experimenter-absent buzzer task*, we detected an effect of condition on the gazing behavior [LRT: $\chi_{\left(3\right)}^{2}$ = 18.06, *p* < 0.001]. This effect was independent of the test session as no condition × session interaction was observed [LRT: $\chi_{\left(3\right)}^{2}$ = 1.36, *p* = 0.715]. Dogs gazed more to their partner's enclosure in the FC and RI conditions compared to the baseline ET condition (see Figure 4, Tables 3, 7). Gazing toward the partner's enclosure was not linked to the number of buzzer presses in the FC condition, but dogs that gazed more toward their partner in the RI condition also stopped pressing the buzzer earlier (Spearman correlation: FC: *r*_*s*_ = −0.30, *p* = 0.160, RI: *r*_s_ = −0. 61, *p* = 0.002). Based on these gazing behaviors, no reaction to inequity could be observed.

### Tolerance test

As no task context × condition interaction could be detected for both behaviors coded during the tolerance test [LRT: co-feeding: $\chi_{\left(3\right)}^{2}$ = 1.13, *p* = 0.769, feed alone: $\chi_{\left(3\right)}^{2}$ = 0.87, *p* = 0.833], we analyzed the data from both versions of the task combined. We found no indication of an effect of task context [LRT: co-feeding: $\chi_{\left(3\right)}^{2}$ = 0.01, *p* = 0.910, feed alone: $\chi_{\left(3\right)}^{2}$ = 1.10, *p* = 0.314], or condition on co-feeding or feeding alone during the tolerance tests [LRT: co-feeding: $\chi_{\left(3\right)}^{2}$ = 3.54, *p* = 0.315, feed alone: $\chi_{\left(3\right)}^{2}$ = 1.26, *p* = 0.739]. Likewise, no effect of test session was observed on these tolerance behaviors [LRT: co-feeding: χ^2^_(3)_ = 1.83, *p* = 0.176, feed alone: $\chi_{\left(3\right)}^{2}$ = 0.86, *p* = 0.352]. This indicates that the dogs did not adjust their co-feeding behavior according to the previous test condition.

## Discussion

The aim of the current study was to investigate the function of the experimenter in an inequity paradigm. In order to explore this, we ran an inequity task, once involving an interaction with an experimenter, who was treating the dogs unequally (*experimenter-present version)*, and once removing this social aspect, having dogs interact with an apparatus that delivered food unequally (*experimenter-absent version*). Contrary to our predictions, we found that dogs did not react to unequal treatment in this paradigm, neither when an experimenter was responsible for inequity nor when a “non-social” setup was used. Nonetheless, their behavior differed in some important aspects, showing that dogs responded to the task differently when the experimenter was visible and actively involved.

In the *experimenter-present* version of the buzzer task, dogs stopped pressing the buzzer when they were not being rewarded (reward inequity, subject receives nothing but partner is given a high value reward), but they stopped to the same extent when no partner was present and the subject, likewise, received no reward (asocial no-reward condition). This indicates that the inequity in reward outcome was not the key variable affecting the dogs' behavior, as the presence of a better-rewarded partner did not change their performance. Rather it is likely that the key variable here is presence or absence of an expected reward regardless of the social setting, consistent with extinction theory. In addition, they did not show the classic “primate” reaction of an earlier refusal to work in the quality inequity (i.e., subject receives low but partner a high value reward) compared to the equity condition (similar to Range et al., 2009; Brucks et al., 2016). Nonetheless, dogs gazed more toward their partner in the reward inequity condition than in the equity condition, also tending to look more when the partner received a reward of higher quality (see Table 8 for summary of results). We can rule out the possibility that dogs were following the movement of the high quality reward rather than monitoring the partner's payoff because firstly, the dogs looked toward their partner less when the high value reward was moved into the partner's enclosure without inducing inequity (as in the food control condition). Secondly, the dogs that gazed more toward their partner, thereby increasing their potential to monitor the partner's payoff, also stopped pressing the buzzer earlier in the inequity conditions than dogs that gazed toward their partner less. Furthermore, the dogs were more stressed when they witnessed that their partner was rewarded. Taken together, the gazing and stress behavior patterns suggest that the dogs were able to detect the inequity but had difficulties in responding to this in a more overt manner by refusing to continue pressing the buzzer. This may be due to dogs' poor inhibitory control; refusing food (any food, whatever the condition) can be especially taxing for them (e.g., Bray et al., 2013; Marshall-Pescini et al., 2015; Brucks et al., in revision). Nevertheless, the overall behavior of the dogs (i.e., in terms of buzzer presses and tolerance behavior following the test) was not consistent with an aversion to inequity in this task. A potential alternative explanation is that the dogs' behavioral pattern in the experimenter-present version of the buzzer task was driven by individual frustration. However, the results are not entirely consistent with this view either, as dogs did not react to the frustrating food control condition and seemed to pay attention to their partner more during the conditions in which they were rewarded unequally.

**Table 8:** **Summary of effects in experimenter-present and -absent version of the buzzer task**.

**Variable**	**Experimenter-present version**	**Experimenter-absent version**
Trials completed	equity > reward inequity	equity > reward inequity; equity > contrast; asocial no-reward < reward inequity
Stress behaviors	equity > reward inequity; equity > contrast; asocial no-reward > reward inequity	
Gaze to partner's enclosure	equity < reward inequity; equity < quality inequity^x^	equity < contrast; equity < reward inequity

x *Only tendency*.

In the *experimenter-absent version* of the buzzer task, dogs stopped pressing the buzzer earlier in the reward inequity condition, and the food control condition, compared to the baseline equity condition, while they did not show any reaction to quality inequity. In addition, they gazed more toward their partner, and showed more stress signals, in the reward inequity but also in the food control condition (see Table 8 for a summary of results). While those dogs that monitored their partner for a longer time refused to press the buzzer earlier in the reward inequity condition, no correlation was found between the gazing behavior in the food control and the equity condition. This indicates that the dogs looked at their partner in unexpected situations (e.g., when receiving no reward or a reward of lower quality than expected) but were getting frustrated when their individual expectations were not being met (similar to squirrel monkeys Talbot et al., 2011, and cottontop tamarins Neiworth et al., 2009). Interestingly, dogs were more inclined to work, but also more stressed, when their partner was present compared to when they were tested alone. This suggests that, in addition to the frustration/contrast effect, dogs were also affected by social facilitation, as the partner dog's presence enhanced the propensity of the subject to continue working although no reward was obtained (see also Dubreuil et al., 2006; Hopper et al., 2013). In line with this, the social tolerance of dogs toward their partner in the food tolerance test was not affected by the treatment in the preceding test condition, as dogs based their refusal on their own expectations rather than comparing it with their partner's outcome in the *experimenter-absent* buzzer task.

These results do not support our predictions, as we could not elicit a clear response to inequity using the current experimental setup. Nonetheless, removing the experimenter from the setup seems to cause dogs to base their refusals on individual reward expectations rather than socially comparing rewards, hence, indicating that the experimenter seems to play some role in inequity tasks. Studies in humans have shown that the intentionality of reward distributions (Blount, 1995; Hachiga et al., 2009; Sloane and Premack, 2012) plays a major role in the rejection rate of unequal offers. If an offer is not attributable to a person and is only caused by a non-social or chance event, people will show no self-interest because no normative expectations are violated (Blount, 1995). Given that the buzzer paradigm did not elicit inequity aversion in the first place, we cannot draw any conclusion about the exact role of the experimenter; however, the dogs behaved differently when an experimenter was causing the inequity compared to the *experimenter-absent* buzzer task in which no experimenter was visible. In combination with earlier results, which revealed that dogs show a differential effect toward the experimenter depending on test condition (i.e., avoidance of the experimenter after unequal treatment in terms of quantity and quality, Brucks et al., 2016), we can cautiously suggest that the experimenter plays a central role in eliciting inequity aversion since, in his/her absence, dogs based their behavior on individual expectations. Nonetheless, it is important to point out that we manipulated not only the presence of the experimenter but also removed the entire interaction between experimenter and dogs in the experimenter-absent task; hence, it would be interesting to investigate how dogs' perception of the task differs if only the interaction component is manipulated (i.e., experimenter is present but not interacting with dogs).

Nonetheless, these results raise the question of why this paradigm could not elicit inequity aversion, a behavior, which dogs have shown in other studies using a paw-giving paradigm (Range et al., 2009; Brucks et al., 2016). The test procedure for the buzzer task differed from the previously used paw task in two aspects: firstly, there was no direct interaction with the experimenter (paw task: give paw in hand palm; vs. buzzer task: placing paw on top of the buzzer). And secondly, the dogs did not directly interact with the experimenter when they received the reward (paw task: receiving reward from the hand vs. buzzer task: receiving reward from two bowls). As previously demonstrated, differences in the experimental setup can strongly affect the animals' reaction to inequity (i.e., rewards for token exchange e.g., Brosnan and de Waal, 2003; Brosnan et al., 2010a vs. rewards for “free” e.g., Bräuer et al., 2006; Roma et al., 2006). In the current study, like in other setups, the dogs were placed directly next to each other and had sufficient visual contact to observe each other during the test. Also, we ensured that dogs understood the task (i.e., pressing the buzzer) through careful training, as seen in the low refusal rates in the rewarded conditions. Furthermore, in a recent study using the same buzzer paradigm with pack living wolves and dogs, dogs showed an aversion to inequity (Essler et al., submitted). More specifically, dogs stopped pressing the buzzer when they were not rewarded and did so earlier when they saw their partner getting a reward compared to the asocial condition, in which no partner was present. In addition, they tended to stop pressing the buzzer when they received a reward of lower quality than their partner.

In most respects, the two procedures were identical; however, two, potentially crucial, aspects differed. Firstly, in the study by Essler et al. (submitted) animals were tested by their trainer/care-giver; hence, they had an established, positive relationship with the experimenter. Given that familiarity has an impact on dogs' motivation to work (e.g., Prato-Previde et al., 2008; Cunningham and Ramos, 2014), it might be the case that the dogs worked longer for their familiar trainer, especially in the non-rewarded condition, due to a positive training history with them. In order to rule out this familiarity explanation, we ran an additional control condition, in which we re-tested dogs previously tested with the experimenter but this time with their owners giving the buzzer commands and rewarding them (see Supplementary Material for details of procedure and results). We found that, even with their owners, dogs did not react to the unequal treatment. The second crucial aspect that was different between the current buzzer task and the study by Essler et al. (submitted) is the fact that, in the latter study, the animals were rewarded by hand from a single shared bowl. Accordingly, this leaves only one explanation for the lack of response to inequity in the buzzer task paradigm: the reward delivery method.

Most studies utilizing an exchange task to investigate inequity aversion stored the rewards in two bowls, with the bowls differing in reward quality, in front of the animals. The rewards were handed out from both bowls (depending on the test condition) by the experimenter (e.g., Brosnan and de Waal, 2003; Brosnan et al., 2005; Fontenot et al., 2007; van Wolkenten et al., 2007). In order to facilitate the reward visibility and traceability for the dogs, we deliberately chose to deliver the rewards in separate bowls. Using one or two shared bowls instead of separate bowls for each individual is likely to increase the competition between partners. Consequently, the reward delivery method we chose may have led dogs to perceive the task more as an individual task reducing the competition with the partner for rewards. Given that each dog had its own set of baited bowls in front of the enclosure, they may have worked primarily to gain access to “their” bowls instead of monitoring and keeping track of the partner's outcome as well. Additionally, in Essler et al.'s study the dogs were rewarded by giving the reward directly out of the hand instead of using bowls, thus, dogs may have perceived the task as more cooperative since, in addition to the verbal and gestural buzzer command, the subsequent reward delivery was more socially interactive than in our experimenter-present task, in which the reward was delivered via bowls. Given that inequity aversion has been proposed to act as a mechanism to stabilize cooperation (Fehr and Schmidt, 1999; Brosnan, 2011), it may be necessary to create a situation that is perceived as cooperative (i.e., the goal should be shared) in order to induce inequity aversion. In addition, inequity aversion should be relevant only in a social context (i.e., dyadic interactions between subjects (e.g., bar-pulling paradigm, Cronin and Snowdon, 2008; Massen et al., 2012), triadic interaction with an experimenter (e.g., token exchange paradigm, Brosnan and de Waal, 2003; Brosnan et al., 2010a) according to the hypothesized link between inequity aversion and cooperation (Brosnan, 2011). Consequently, paradigms investigating inequity aversion might involve two important aspects: on the one hand, animals are competing with the partner for the rewards; however, they are also potentially cooperating with the experimenter in the case of token-exchange paradigm or directly cooperating with a partner in the bar-pulling paradigm to obtain rewards. If one of those requirements is not fulfilled (i.e., lack of a competitive component in buzzer paradigm, lack of cooperative component in paradigms that do not involve investment of effort, e.g., Dubreuil et al., 2006; Roma et al., 2006), the paradigm may not elicit inequity aversion. In fact, the addition of the visible experimenter in the *experimenter-present* buzzer task may have caused dogs to perceive the task as a more cooperative one, explaining their increased sensitivity to the partner's outcome (e.g., increased gazing and stress behaviors); however, the lack of a competitive component (shared bowl) may have limited the extent to which the subject perceived and reacted to the unequal treatment. Consequently, future research will be needed to elucidate the importance of cooperation/competition for regulating the response to unequal treatment in inequity paradigms. This could be investigated, for example, by using a paradigm that is known to elicit inequity aversion, such as the paw-giving paradigm for dogs (e.g., Range et al., 2009) or the exchange paradigm for primates (e.g., Brosnan and de Waal, 2003), and testing how individuals react to unequal treatment if rewards are delivered and stored in separate bowls, one set per individual.

In conclusion, we suggest that a social and potentially cooperative interaction with either an experimenter or directly with a partner is needed in order to elicit inequity aversion; however, a competitive element may also be necessary. Future research is needed to understand the effects of cooperation and competition in governing inequity aversion. In addition, it remains to be seen whether similar results are found in other species or whether the unique relationship between dogs and humans makes the interaction with the experimenter more significant for dogs than for other species.

## Author contributions

DB, SM, JE, FR designed the experiments. DB, JE, JM conducted the experiments. DB and JE coded videos. DB analyzed the data. DB, SM, JE, JM, LH, and FR wrote the paper.

### Conflict of interest statement

The authors declare that the research was conducted in the absence of any commercial or financial relationships that could be construed as a potential conflict of interest.
