# Supplementary material for: What Are the Ingredients for an Inequity Paradigm? Manipulating the Experimenter's Involvement in an Inequity Task with Dogs
Source: Front Psychol. 2017 Feb 28;8:270. doi: 10.3389/fpsyg.2017.00270 (PMC5329037; doi:10.3389/fpsyg.2017.00270)
Supplement: Supplementary file 2 [file SupplemetaryInformation.DOCX]

**Supplementary Material**

**Movie 1.** Clip showing test procedure for experimenter-absent and experimenter-present version of the buzzer task.

**Table S1.** Individual characteristics of dogs (*N* = 44), test group and low value rewards (LVR) per dyad

| Dyad | Name | Sex | Breed | Age (yrs.) | Buzzer task | LVR |
| --- | --- | --- | --- | --- | --- | --- |
| 1 | Achuk | F | Chesapeake Bay Retriever | 8.3 | exp-absent | dry food |
|  | Elrond | M | Chesapeake Bay Retriever | 4.6 | exp-absent | dry food |
| 2 | Aiko | M | Australian Shepherd | 2.4 | exp-absent | dry food |
|  | Emely | F | Bernese Mountain Dog | 1.7 | exp-absent | dry food |
| 3 | Baja | F | Australian Shepherd | 1.1 | exp-absent | dry food |
|  | Daimony | F | Australian Shepherd | 6.7 | exp-absent | dry food |
| 4 | Bessy | F | Border Collie | 2.6 | exp-absent | dry food |
|  | Eve | F | Border Collie | 6.0 | exp-absent | dry food |
| 5 | Bella | F | Bernese Mountain Dog | 6.5 | exp-absent | cheese |
|  | Brandy | F | Bernese Mountain Dog | 3.0 | exp-absent | cheese |
| 6 | Cole | M | Border Collie | 6.4 | exp-absent | dry food |
|  | Esprit | F | Border Collie | 3.7 | exp-absent | dry food |
| 7 | Emily | F | Border Collie | 5.7 | exp-absent | carrot |
|  | Ziva | F | Border Collie | 2.1 | exp-absent | carrot |
| 8 | Geischa | F | Australian Shepherd | 8.1 | exp-absent | dry food |
|  | Yuuki | M | Australian Shepherd | 2.1 | exp-absent | dry food |
| 9 | Luke | M | Border Collie | 8.0 | exp-absent | dry food |
|  | Quismo | M | Border Collie | 6.1 | exp-absent | dry food |
| 10 | Luna | F | Siberian Husky | 1.2 | exp-absent | cheese |
|  | Talie | M | Siberian Husky | 2.8 | exp-absent | cheese |
| 11 | Mago | M | Golden Retriever | 9.1 | exp-absent | carrots |
|  | Tika | F | Husky - Mix | 6.1 | exp-absent | carrots |
| 12 | Sokrates | M | Bardino – Mix | 7.9 | exp-absent | dry food |
|  | Ultimo | M | Border Collie | 4.2 | exp-absent | dry food |
| 13 | Age | M | Malinois | 8.3 | exp-present | dry food |
|  | Balthasar | M | Hollandse Herder | 1.8 | exp-present | dry food |
| 14 | Akin | M | Rhodesian Ridgeback | 6.9 | exp-present | carrot |
|  | Zuri | F | Rhodesian Ridgeback | 6.3 | exp-present | carrot |
| 15 | Akira | F | Irish Setter-Mix | 9.4 | exp-present | dry food |
|  | Charissma | F | Weimaraner | 8.0 | exp-present | dry food |
| 16 | Bela | M | Australian Shepherd | 1.0 | exp-present | dry food |
|  | Spicy | M | Australian Shepherd | 5.8 | exp-present | dry food |
| 17 | Chilli | F | Australian Shepherd | 7.8 | exp-present | lettuce |
|  | Linus | M | Australian Shepherd | 1.8 | exp-present | lettuce |
| 18 | Cira | F | Siberian Husky | 6.4 | exp-present | dry food |
|  | Cotya | M | Siberian Husky | 6.4 | exp-present | dry food |
| 19 | Clio | F | Mixed breed | 1.9 | exp-present | dry food |
|  | Lola | F | Border Collie - Mix | 3.3 | exp-present | dry food |
| 20 | Clooney | M | Border Collie | 7.3 | exp-present | dry food |
|  | Jamie | M | Border Collie | 3.8 | exp-present | dry food |
| 21 | Loomie | F | Pitbull - Mix | 2.4 | exp-present | dry food |
|  | Mia | F | Smooth Collie | 5.8 | exp-present | dry food |
| 22 | Hybie | F | Labrador - Mix | 7.2 | exp-present | cucumber |
|  | Tuukka | F | Mixed breed | 2.1 | exp-present | cucumber |

**Food preference test**

In order to establish two reward types of different quality for each dyad we conducted a food preference test with each dog prior to testing. Fist, we asked the owner, which type of food their dogs do not like in particular but would still eat and work for (LVR) and which reward their dogs particularly like (HVR). Both dogs in a dyad received the same food rewards (see Table S1 for rewards used for dyads).

In the subsequent test, we confirmed those suggested reward preferences by repeatedly presenting one dog at a time both reward types simultaneously. The experimenter kneeled in front of the dog, a distance of 2 m away and presented one piece of each reward type on two different coloured lids (black and white) by leaning towards the dog. The colour on which the HVR was presented was counterbalanced across dyads. The dog was restrained by the owner who was sitting on a chair. After the dog had sniffed both rewards, the experimenter placed the lids equidistant from the dog on the floor with a distance of 60 cm between the lids and then removed her hands. The dog was then released and allowed to eat one reward. The lid that was not chosen was removed immediately. If a dog directly approached one lid and ate the reward, it was considered a choice. After the dog finished eating the reward, the owner called them back and the next trial started. The side of rewards was alternated between trials but counterbalanced across sessions. Twelve trials were conducted per session and if a dog chose the HVR in 9 trials, this was considered a preference (binomial: *p* = 0.02). If this criterion was not met, another session was conducted following the same procedure. However, if no preference was found within three sessions, other reward types were chosen and tested in the same way.

**Training procedure**

The dogs’ training for the buzzer task involved two steps. Firstly, dogs were trained to associate the reward types with each bowl colour and secondly, dogs learnt to press a buzzer to receive a reward. Training was conducted on two consecutive days. To train dogs to associate food type to bowl colour, the previously established food types (LVR and HVR from the preference test) were placed in two bowls, identical in size (ca. 10 cm diameter each) but differing in shape and colour (black & rectangular vs. white & round). Bowls were attached to a 1 m stick, so the experimenters could move them in and out of the enclosures while hidden behind the curtain. Bowl colour and food type association was counterbalanced across dyads. The owner sat on a chair and held the dog on a leash in front of him/her. One dog was trained at a time while the other dog was leashed at the opposite end of the room. The experimenter, while kneeling on the floor (a distance of 2 m from the dog), visibly baited both bowls with one piece of the corresponding reward type. After doing so, the bowls were moved towards the dog (within 30 cm of the dog’s nose) using the sticks attached to the bowls. The dog was allowed to sniff both bowls for 2 sec. while, using the leash, the owner restricted him from taking the reward. Then, the bowls were moved to a distance of 80 cm from each other but equidistant to the dog (80 cm distance to dog), the experimenter looked down and the owner dropped the leash, allowing the dog to make a choice. As soon as the dog touched one bowl, the other bowl was moved back again and the choice was noted. After eating the reward, the dog was called back and both bowls were re-baited. Twelve trials were conducted alternating the side of rewards. The criterion was set at 9 HVR choices (binomial *p* < 0.02) and if the criterion was not met, another session was conducted directly after the first one.

In the subsequent training step, the bowls were still visibly baited but not pushed as close to the dog (bowl movement stopped at a distance of 1 m distance from the dog) and the distance between bowls was increased (1.5 - 1.7 m between bowls). With this step we wanted to test whether dogs had learnt to discriminate between bowls based only on the bowl colour and shape. Following the same procedure as for the previous step, 20 trials were conducted in 2 sessions on two different days (see Movie 1 for training procedure). To move on to the next training step, we set the criterion at 17 HVR choices (binomial: *p* < 0.001) in two consecutive sessions. If a dog did not reach the criterion, the session was repeated; however, if dogs did not reach criterion within 5 sessions, the dyad was excluded. No more than 2 sessions were conducted per day.

Following the association trials, we trained the dogs to press the buzzer with their paw. This was done using positive reinforcement using only LVR and verbal praise as rewards. One dog was trained at a time. In the first training step, the dogs were rewarded for placing their paw on top of the buzzer, which was held by the experimenter. For the non-social buzzer task, no command was given, whereas a specific command (e.g. ‘paw’, ‘press’, ‘step’, etc.) was introduced at this point for the social version. On the next test day the second training step was conducted, again training one dog at a time. To start with, a few trials of the first training step were repeated, to remind dogs of the task. Then the buzzer was positioned on a wooden block (see below for more information). The experimenter moved the buzzer towards the dog and remained silent (non-social version) or gave the specific command (social version). If the dog placed their paw on top of the buzzer they were rewarded, whereas if it did not, the experimenter went a step back in training. If the dog pressed the buzzer reliably for 5 trials without further cuing, it was introduced to the test enclosure. While sitting inside the test enclosure the dog was presented with the buzzer through the fence and reinforced for pressing it (i.e. LVR tossed inside of enclosure). Note that the owner was still visible and the experimenter was sitting visibly in front of the enclosure for both tasks. If the dog pressed the buzzer in 5 consecutive trials without any additional cues and commands (experimenter-absent) being given by the experimenter, the owner moved behind a visual barrier. For the experimenter-absent version of the buzzer task, the experimenter now also moved behind the curtain, whereas in the experimenter-present task, the experimenter remained in front of the curtain. Again, the buzzer was moved inside and if the dog pressed it, a reward was tossed inside the enclosure from behind the curtain. No more praising words were used at this point, and in the case of the non-social version, no more commands were issued. If the dog failed to press the buzzer after it was moved into the enclosure, the experimenter moved back in front of the curtain and rewarded the dog from this position before moving behind the visual barrier again (experimenter-absent version) or the experimenter cued the dog more to the buzzer by giving the pointing gesture closer to the buzzer (experimenter-present version). In the final step, a plate containing pieces of LVR was made visible, but not accessible, before the buzzer was moved inside the enclosure to familiarize dogs with the presence of food during the test. As before, the buzzer was slid into the enclosure and after the dog successfully pressed it, one piece of LVR was tossed into the enclosure (see Movie 1 in supplementary methods for training steps). Training was completed, when the dog pressed the buzzer without any commands (experimenter-absent version) and/or additional cues (experimenter-present version) for 15 times in a row (i.e. 5x rewarded, 5x not rewarded, 5x rewarded). If a dog did not reach this criterion, another training session was conducted on another day.

**Table S2.** Definitions of stress behaviours coded during the inequity task and their average occurance during each test trial combining both versions of the buzzer test.

| **Variable** | **Description** | **Average per trial** |
| --- | --- | --- |
| Yawn | Dog widely opens jaws | 0.05 ± 0.01 |
| Scratch | Dog scratches body with either paw or nibbling with mouth | 0.01 ± 0.00 |
| Mouth-liking | Dog extrudes tongue from mouth and runs it over the lips | 0.25 ± 0.02 |

**Control for effect of experimenter’s familiarity**

*Procedure*

Five dog dyads familiar with the buzzer paradigm participated in this owner control experiment. The previous test and this control experiment were carried out, on average 12.3 ± 4.4 months apart. Three of these dyads had participated in the experimenter-present and two dyads in the experimenter-absent version of the buzzer task. The test procedure was the same as that of the experimenter-present version of the buzzer task. The experimenter instructed the owner to point towards the buzzer while giving a verbal command and to slide the food bowls into the enclosures. In order to provide the owners with the opportunity to familiarise themselves with the test procedure, and to re-familiarise the dogs with the task, 15 training trials were conducted before the first test session. In these trials, only one dog was in the enclosure; the owner asked them to press the buzzer when it was made available and rewarded them dog for doing so. The rewards were delivered on a plastic plate rather than the delivery bowls used in the test, as was the case during the normal warm-up trials, The dogs were rewarded with a piece of low-value reward for pressing the buzzer. The same rewards were used for each dyad as was the case during the initial test phase. All dogs readily completed the training trials without any need for further training. Following the training phase, all dogs had a ten-minute break in the test room, in which they were allowed to move freely before the first test condition started. To reduce the likelihood of habituation effects (since these dogs had already been tested in the past), dogs were tested only in three crucial conditions i.e. equity (ET), reward inequity (RI) and non-social no reward (NR) conditions. Each dog served as the subject and the partner and the order of test conditions was counterbalanced and randomised across dyads. Two test conditions were conducted per test day with at least a seven minute break in-between each condition.

*Results*

The number of buzzer presses differed between conditions (Friedman: *χ^2^* = 11.24, *df* = 2, *N* = 10, *p* = 0.004; Fig. S1). Dogs pressed the buzzer less often in the RI condition compared to the ET condition (Wilcoxon Test: *T* = 0, *N* = 10, *p* = 0.009). There was no difference in the number of times the buzzer was pressed between NR and RI condition (Wilcoxon Test: *T* = 14.5, *N* = 10, *p* = 0.373). Session order (e.g. 1, 2, 3 or 4^th^ session) did not influence dogs’ performance across conditions (Kruskal-Wallis Test: ET: *df* = 3, *p* = 0.506; RI: *df* = 3, *p* = 0.901 and NR: *df* = 3, *p* = 0.086).

**Figure S1.** Number of times the buzzer was pressed when prompted by the owners across test conditions (*N* = 10 dogs). ET = equity, RI = reward inequity, NR = no-reward control condition.
